# Handling Diagnosis of Schizophrenia by a Hybrid Method

**DOI:** 10.1155/2015/987298

**Published:** 2015-03-02

**Authors:** Luciano Comin Nunes, Plácido Rogério Pinheiro, Tarcísio Pequeno Cavalcante, Mirian Calíope Dantas Pinheiro

**Affiliations:** Graduate Program in Applied Informatics, University of Fortaleza (UNIFOR), Avenida Washington Soares, 1321, Bl J Sl 30, 60811-905 Fortaleza, Brazil

## Abstract

Psychotics disorders, most commonly known as schizophrenia, have incapacitated professionals in different sectors of activities. Those disorders have caused damage in a microlevel to the individual and his/her family and in a macrolevel to the economic and production system of the country. The lack of early and sometimes very late diagnosis has provided reactive measures, when the professional is already showing psychological signs of incapacity to work. This study aims to help the early diagnosis of psychotics' disorders with a hybrid proposal of an expert system that is integrated to structured methodologies in decision support (multicriteria decision analysis: MCDA) and knowledge structured representations into production rules and probabilities (artificial intelligence: AI).

## 1. Introduction

Psychological disorders have been identified among the main causes of absence from work environments, in view of the influence that those disorders may have on the immune system of people, generating diseases to them. However, some factors hinder a proper diagnosis; for example, a person suffering from psychological disorder offer resistance to accept such a situation, while a healthy person can simulate some symptoms, in an attempt to circumvent a situation to their advantage [1].

In the set of psychological disorders, the psychotic disorders are more incapacitating, characterized by behaviors as those at schizophrenia. A terrible consequence for an individual who is suffering from psychotic disorders is the image of invalidity that the individual presents for the society in which he lives. By more than the, individual strives to erase the negative image formed in respect of his person, the prejudices and the damage are difficult to reverse.

It is usually observed in the workplace as people react with a teammate who suffers from a psychotic disorder. Even if renowned experts are treating him or he/she is ingesting drugs of last generation, many of his work team observes him/her with suspicion. A mutilation of a limb, a serious infection controlled by medication, and even a cancer eradicated can provide chances for reinstatement of an individual to social life and to the work. However, the stigma of being a carrier of a psychotic disorder usually invalidates the professional who suffers from it. Aiming at the propose of a system for early diagnosis of psychotic disorders, a hybrid model is presented by combining multicriteria methodology for decision support and expert system. Importantly, hybrid models have been used to support decision-making and finding diagnostics for diseases [2–5].

Considering the greater organicity proportioned as well as being a version of the experience of mental health professionals, it was decided to use the model of the fourth edition of the* Diagnostic and Statistical Manual of Mental Disorders*,* DSM-IV*, recommended by the American Psychiatric Association. Also, in the study relations of forces between symptoms and causes of psychotics disorders were established, in order to form a classification, aiming at formatting rules to be used in knowledge base of expert system to assist in establishing the desired diagnostic process.

## 2. A Methodology for Classification of Diseases

### 2.1. Diagnostic and Statistical Manual of Mental Disorders, Fourth Edition

As previously mentioned, this study was based on the fourth edition of the* Diagnostic and Statistical Manual of Mental Disorders* (*DSM-IV*); due to this manual, much is already known in the area of mental health, in several parts of the world, and thus, it itself constitutes a reference book for many. The* DSM-IV* describes typical patterns of behavior, thought, and emotion, allowing categorizing the diagnosis. The categories are prototypes. A patient who is suffering events related to the prototype is said to be having the disorder relating to that prototype. Qualifiers are sometimes used, to moderate or severe form of the disorder. Each category of disorders has a numeric code that is used by providers of health services for administrative purposes. The structure of the* DSM-IV* is axial; that is, it shows the diagnostic types of mental disorders arranged in five axes, as listed below:

- Axis I: clinical disorders, mental disorders, developmental disorders, and learning disorders are included here;
- Axis II: personality disorders and mental retardation;
- Axis III: medical conditions or acute physical disorders;
- Axis IV: psychosocial or environmental factors that are correlated with the disturbances;
- Axis V: global assessment of function (global assessment of functioning) or global assessment scale for children (children's global assessment scale) for youth under the age of 18 (on a scale of 0 to 100).

### 2.2. A Classification of Diseases Applied to Psychological Contextualization

**Figure 1:**
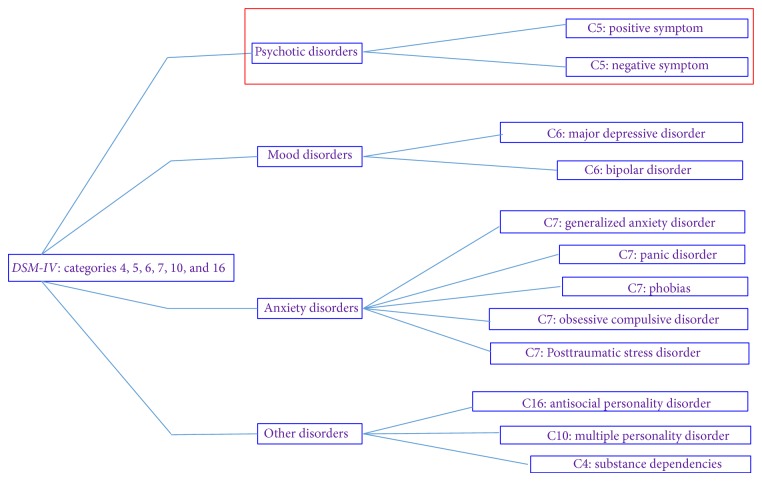
Psychological disorders. Source: formatted by the author from HiView software.

**Figure 2:**
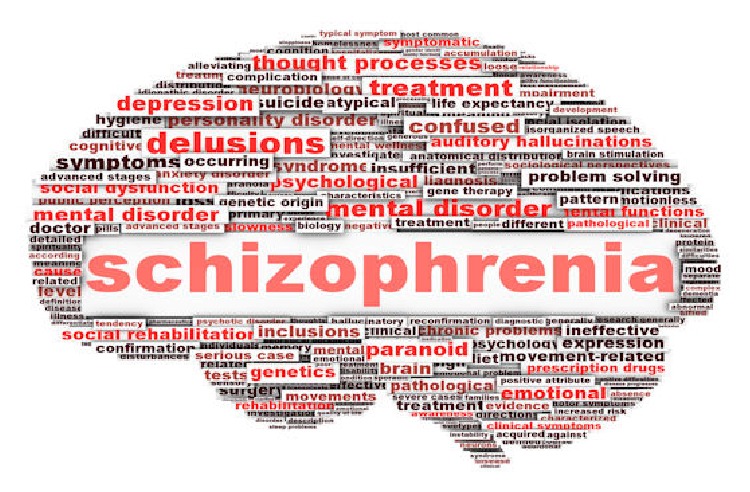
Psychotic disorders. Source: http://www.shutterstock.com

Based on the premise that it is possible to identify causes and symptoms to psychotic disorders, we seek early diagnosis of these disorders. To this end, in this study related “control events” that correlate with symptoms and causes that show mentioned psychotic disorders were presented [5]. For each of these “control events” that appear in psychotic disorders, a confidence factor that indicates the degree of influence on the outcome of the indicative diagnosis of these disorders is assigned. The tree shown in Figure 1, formatted using the HiView software, summarizes the major psychological disorders that affect people. Specified disorders represent some classes presented in the* DSM-IV*, among which the category of psychotic disorders stands out in Figure 2, subject of this paper.

### 2.3. Psychotic Disorders

More commonly known as schizophrenia, term of Greek origin, that means “split mind,” for the individual who suffers this disease there is dissociation between his/her thinking and the reality in which he/she lives. Thus, the individual with this psychological disorder acts as if he lived in a world that exists only in his mind, disconnected from reality. Psychotic disorders are considered the most severe and disabling form of mental disorders. These disorders usually emerge in adolescence or early adulthood and rarely after 45 years of age [1]. For this type of psychological disorder, in this paper itself,* DSM-IV* (*Diagnostic and Statistical Manual of Mental Disorders, Fourth Edition*) is used, which contemplates two groups: positive symptoms and negative symptoms. Many experts and scholars have proposed this system of categorization into two groups of schizophrenia on the subject, among which worth mentioning [7–11]. The* DSM-V* version, released by the American Psychiatric Association (APA) in May 2013, which presents a different categorization for this disease, will be used in future works.

#### 2.3.1. Positive Symptoms Psychotic Disorders

This disorder type's main characteristics are as follows [1]:

- Frequent manifestations of delusions or bizarre thoughts, that is, outside the sociocultural context of the individual, and as much as it itself argues against using including corroborative veracity of the delirium's elements, the individual who suffers from this disorder does not accept the explanations;
- hallucinations or sensory perceptions without the presence of a being or object relative to the manifested sense, such as voices and sounds directed only to the individual who is suffering from the disorder;
- laughing and crying easily, without explanation;
- manias to be suffering persecution;
- disorganization of thought, barring participation in dialogues or expressions of related ideas.

**Figure 3:**
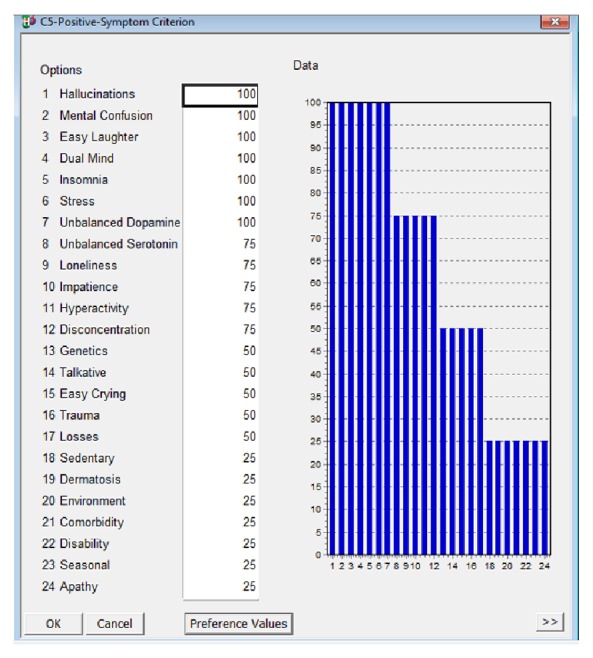
Events correlated psychotic disorders positive symptoms. Source: formatted by the author from HiView software.

In Figure 3, the main control events, which correlate with psychotic disorders positive symptoms, are provided. The numerical values in Figure 3 represent the importance of each event in the occurrence of this disorder; that is, the higher the value is, the more influence this event has in establishing the diagnosis for this disorder. These values were assigned based on a scale of importance of each event to the occurrence of each disorder. That scale, detailed in Table 1, was created from reports made by experts in the psychiatry and psychology areas, about the degree of importance of the events of control in psychological disorders, object of the present study.

#### 2.3.2. Psychotic Disorders Negative Symptoms

People suffering from this disorder exhibit behavior with the following characteristics [1]:

- the search for the life in closure, total isolation, and avoiding social interaction;
- affective flattening, that is, gradual reduction of affective relationships;
- abulia, or loss of willingness to engage in any activities; reaching the extreme situation of staying motionless, the individual eyes are fixed at anything;
- poor thoughts of ideas manifested by monosyllabic vocabulary.

**Figure 4:**
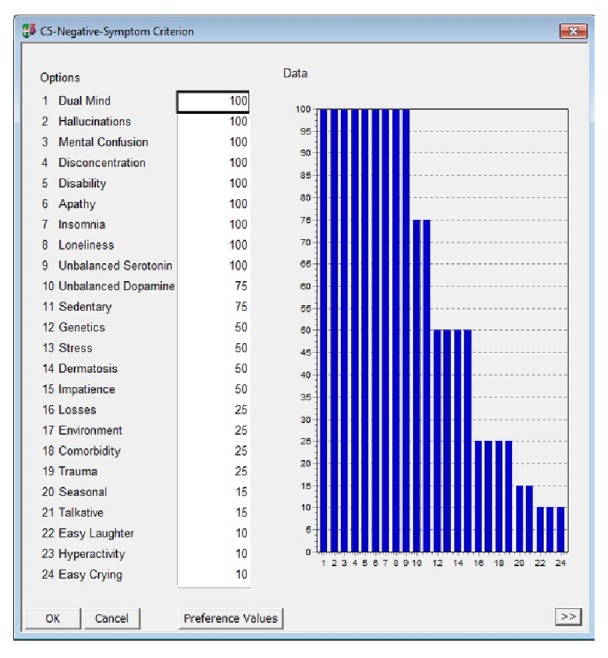
Events correlated psychotic disorders negative symptoms. Source: formatted by the author from HiView software.

In Figure 4, it is possible to observe control events associated with psychotic disorders negative symptoms. In the scale, as shown in Table 1, the numerical values represent the percentage of importance of each event in the occurrence of this disorder; that is, the higher the value is, the greater the influence of this event in establishing the diagnosis for this disease is.

## 3. A Model for Decision Support in Early Diagnosis of Psychotic Disorders

**Figure 5:**
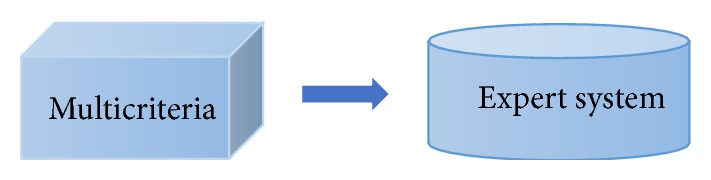
Simplified view of the system architecture, based on a hybrid model of information technologies. Source: formatted by the author.

**Figure 6:**
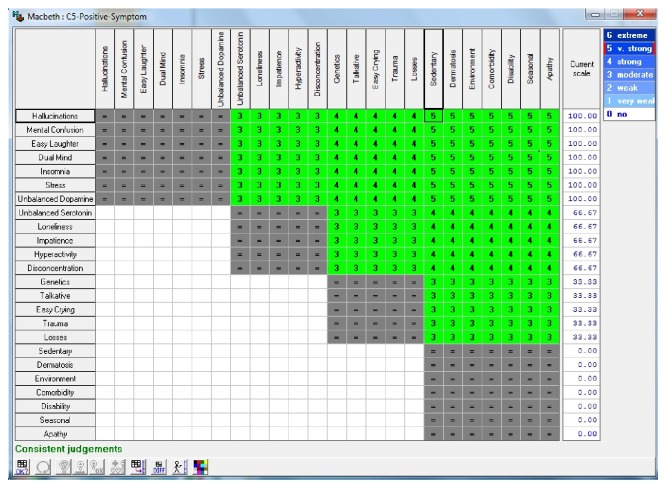
Matrix of judgment and values difference in attractiveness between the “control events” of the psychotic disorders positive symptoms. Source: formatted by the author from HiView software.

**Figure 7:**
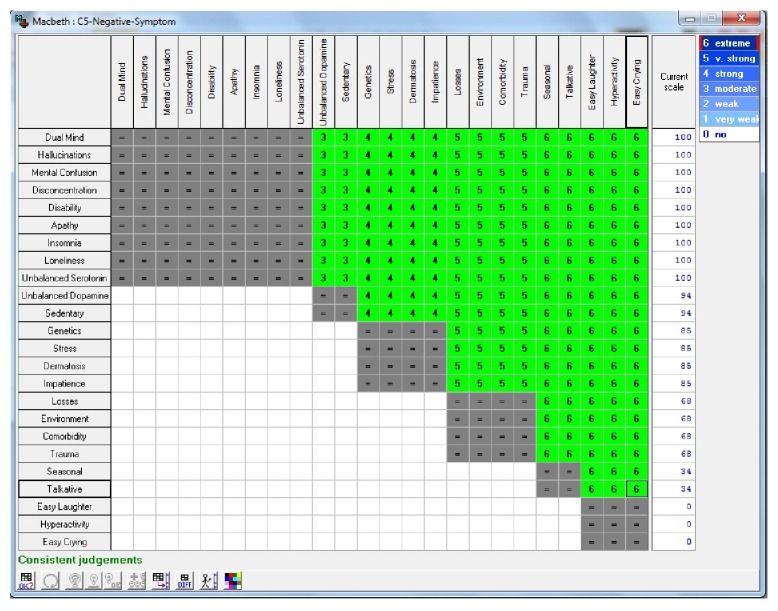
Matrix of judgment and values difference in attractiveness between the “control events” of the psychotic disorders negative symptoms. Source: formatted by the author from HiView software.

This study aims to help the early diagnosis of psychotics disorders with a hybrid proposal of an expert system that is integrated to structured methodologies in decision support (multicriteria decision analysis: MCDA) and knowledge structured representations into production rules and probabilities (artificial intelligence: AI).  Figure 5 presents the flow of this hybrid model of “multicriteria decision analysis” with an “expert system.” Therefore, this study shows that the “control events” and their “confidence factors” listed in Figures 6 and 7 are exported to the expert system, to compose the database of this system and thus aid in the diagnosis of psychotic disorders classified in* DSM-IV* and described in Figures 3 and 4.

### 3.1. Stages in the Process of Decision Support

Main stages of the decision support process, according to [12], are as follows.

- Structuring: it deals with the problem formulation and identification of goals. This phase seeks to identify, characterize, and organize the factors considered important in the decision support process.
- Evaluation: it allows the subdivision on a subphase partial evaluation of actions (alternatives) according to each point of view (criteria) and an overall evaluation considering several partial reviews.
- Recommendation: in this phase sensitivity analyses and robustness are made to verify those changes, in the parameters of the evaluation model, affecting the final result. It is a key phase that helps to generate knowledge about the problem and thus increases confidence in the results obtained from the decision-maker.

### 3.2. Decision Support in the Area of Mental Health

Initially, it would be worth noting that, searching the literature of decision support in health, some studies reported hybrid models used to aid decision-making and finding diagnostics for diseases; among these studies highlight the following: [3, 5, 13, 14].

According to [14], decision analysis is intended to enhance the quality of decisions and communication among physicians, patients, and other healthcare professionals. Unquestionably, many decisions in healthcare are complex, with multiple factors affecting the decision of a particular outcome. It has been heard or has had the experience of some of the deficiencies caused by a decision taken improperly. People of all ages and all states of health have been affected by this type of practice of establishing a diagnosis [15].

It is clear therefore that the decision-making in seeking diagnosis for a health disorder should not be treated informally, practice somewhat common these days, most likely due to the short time spent in search processes diagnosis, especially in the public health service. This line may lead to risks of compromising the quality of diagnosis as well as the health of sick individuals and, consequently, the health system as a whole, generating waste resources and increasing spending. In this trail of problems, it can also raise a culture of self-diagnosis, where lay people self-medicate, because the patients observe that health professionals do not give proper attention to their problems and act by trial error/hit. Therefore, individuals are induced to mimic the health area professionals, through the use of the practice of the error/hit, complicating the search by the diagnostic process.

This work aims, among other things, to show the positive impact of the use of information technology in support-establishing clinical diagnoses of psychotic disorders. Szolovits [16] describes various approaches that try to present a great way of using information technology in healthcare, particularly through systems supporting diagnosis of mental disorders. The motivations for the development of such studies, as also described by Szolovits [17], are readily visible because excellence in health care is of paramount importance to society.

### 3.3. Problems of Decision Support and Methodologies Multicriteria

To establish a diagnosis of psychology and psychiatry, studies are needed, experiments and observations which are aware of the symptoms that lead to the cause of the disorder evidenced. A major difficulty in reaching a correct diagnosis is the complexity of factors evidenced, for, besides the large amount of information, the expert needs to take into account cultural, biological, psychosocial issues, quality of information, signs, and symptoms common to many diseases. This complicates the decision process, turning it into something complex, and it also takes into account unstructured information. The methodology to aid multicriteria decision-making has much to add to the diagnostic process in psychology and psychiatry. In order to put at the disposal of the decision-maker, methods and tools enable them to design the control events in question as well as prioritizing these events for proper classification.

### 3.4. Multicriteria Methodology

#### 3.4.1. Basics Multicriteria Decision Aiding

Problems involving multiple criteria have several agents and concepts, reasoning its definition which is merely didactic [18]. Some of them are discriminated bellow.

Decision-maker agent has the power and responsibility to ratify the decision, assuming the consequences of this act, whether positive or negative. The decision-maker can be an individual or a group of people establishing the boundaries of the problem, specifying the objectives to be achieved and issues opinions. Not all decision-makers have the power of choice. So it is important to distinguish the degree of influence on the decision-makers' process [6, 19].

Analyst is an agent who interprets and quantifies the opinions of decision-makers, conducts the structuring of the problem, formulates a mathematical model, and presents the results to the decision. The analyst has a duty to act in continuous dialogue and interact with decision-makers, in a constant learning process.

Model is a simplified representation or interpretation of reality through rules and mathematical operations that allow transforming preferences and opinions of decision-makers in quantitative result.

Alternative is known as potential action. The subject of the decision or that is directed to support the decision [20]. It is identified early in the decision process or during this and could become a solution to the problem under study [6].

Criterion represents the preferences of the decision-maker under certain viewpoint of him/her. It is presented as a function *g*, defined on a set *A*, which assigns value's sort of set *A* [21].

According to [21], when a problem has multiple criteria, they are defined as *g*_1_, *g*_2_,…, *g*_*j*_,…, *g*_*n*_. The evaluation of an action “*a*” according to the criterion “*j*” is represented by *g*_*j*_(*a*). The representation of different viewpoints (aspects, factors, and characteristics), with the help of a family *F* = {*g*_1_,…, *g*_*j*_,…, *g*_*n*_} of criteria, constitutes one of the most delicate parts in formulation of decision problems.

Relation of dominance is a relationship that occurs when two elements “*a*” and “*b*” belong to the set *A*; “*a*” dominates “*b*” (*aDb*) if and only if *j* = 1,2,…, *n* wherein at least one of the inequalities is preferably narrow. It may be noted that the dominance relation of “*a*” and “*b*” is characterized by being a strict partial order, being an asymmetric relation and transitive. If “*a*” dominates “*b,*” “*a*” is greater than “*b*” in all criteria of the problem [21].

Efficient Action—The action (or alternative) “*a*” is considered efficient if and only if no other action set* A*, dominates. The efficient set of actions *A, where A* maybe the dominance relation is empty and is generally regarded as a set that contains the interesting actions, to be analyzed in greater depth, even though they lack good reasons to disregard the inefficient [21].

Decision matrix termed as the evaluation matrix, where each row explicitly measures evaluations of alternative *i* with respect to *n* criteria considered. Each column in turn expresses evaluations of the measurements of the *m* alternatives with respect to criterion *j*. Assuming that *a*_*ij*_ represents the evaluation of alternative (or action) *A*_*i*_, then belonging to the set of potential actions *A*, ⌊*a*_*ij*_⌋, according to the criterion *g*_*j*_, one could construct a similar matrix to that shown in Table 2 [6].

#### 3.4.2. Multicriteria Decision Aiding

The method MACBETH (Measuring Attractiveness by a Categorical Based Evaluation Technique) is an approach to multicriteria decision support. The research that initiated the method was performed by Antonio Carlos Bana e Costa and JC Vansnick in the early 90s [12]. This methodology has emerged in response to the question of how to build an interval scale of preferences from a set of options without forcing decision-makers to produce their preferences numerically directly. This approach allows you to assign scores to each alternative through a paired comparison. Given two alternatives, the decision-maker must express the most attractive in the case have greater confidence, and what degree this attractiveness on a scale that has semantic correspondence with an ordinal scale. The program itself makes the analysis of cardinal consistency (transitivity) and semantics (relations between differences), suggesting, in the event of inconsistency, how to resolve it. It is still available to the decision-maker to graphically adjust the value of the scores assigned, within permitted ranges. According to [12], the construction of cardinal value scale is only after this adjustment, with the introduction of specialist expertise, which is characterized. The difference of attractiveness is very important in this methodology.

According to [22], the MACBETH method, when the decision-maker is asked to value judgments about potential actions (alternatives) in a given situation are realized, it will do in terms of attraction he feels for this alternative. This task is defined on the construction of the role, criterion *v*_*j*_, such that

- for *a*, *b* ∈ *A*, *v*(*a*) > *v*(*b*), if and only if, for the evaluator, *a* is more attractive (locally) than *b* (*aPb*);
- any positive difference, *v*(*a*) > *v*(*b*), represents the numerical difference in value between “*a*” and “*b*,” with “aPb” being always in terms of a fundamental point of view *j* (*FPV*_*j*_) or criterion “*j*”;
- Then, for each “*a*,” “*b*,” “*c*,” “*d*” ∈ A, being judged a more attractive than “*b*”, and “*c*” more attractive than “*d*”, it is clear that *v*(*a*) − *v*(*b*) > *v*(*c*) − *v*(*d*), if and only if the difference of attractiveness between *a* and *b* is greater than the difference in attractiveness between “*c*” and “*d*”.

For the MACBETH method is important, the following is reasoning. Given the impacts *ij* (*a*) and *ij* (*b*) of two potential actions “*a*” and “*b*,” according to a fundamental point of view FPV_j_, being judged a more attractive than “*b*,” the difference in attractiveness between “*a*” and “*b*” is judged as “null,” “very weak,” “weak,” “moderate,” “strong”, “very strong,” or “extreme.” It introduced a scale formed by different semantic categories in attractiveness; the size is not necessarily equal, to facilitate the interaction between the decision-maker and analyst. The semantic categories, *Ck*, *k* = 1,…, 6, are represented as follows [12]:

- *C*1 is very weak difference of attractiveness (or between zero and weak) →*C*1 = [*s*1, *s*2] and *s*1 = 0;
- *C*2 is weak difference of attractiveness →*C*2 = ]*s*2, *s*3];
- *C*3 is moderate difference of attractiveness (or between weak and strong) →*C*3 = ]*s*3, *s*4];
- *C*4 is strong difference of attractiveness →*C*4 = ]*s*4, *s*5];
- *C*5 is strong difference of attractiveness (or between strong and extreme) →*C*5 = ]*s*5, *s*6];
- *C*6 is extreme difference of attractiveness →*C*6 = ]*s*6, +[.

To facilitate the expression of absolute judgments of difference in attractiveness between the pairs of alternatives, it is useful to construct the arrays of value judgments [23].

Considering one software to support multicriteria decision, the M-MACBETH allows the structuring values of trees, construction of the rating criteria, the development of functions values, the weight of criteria, and extensive sensitivity analyses and robustness on value intrinsic and relative options.

#### 3.4.3. Application of MACBETH Method and Software HiView

In this paper the following concepts, techniques, methods, and tools have been applied. Method MACBETH (Measuring Attractiveness by a Categorical Based Evaluation Technique) was applied in supporting decision-making. It has been chosen because it is a method that has been specializing in the use of hierarchy of importance of components of multicriteria events, printing speed in decision-making, especially in the area of human health. The MACBETH method has been used for defining the existing attractiveness between events of control psychological disorders, to simplify the professional judgment of the decision-maker since the entire set of alternatives does not need to be evaluated simultaneously. It is notable, however, that the difficulty for a decision-maker to remain consistent is, mainly, when the number of alternatives and criteria increases. To circumvent this problem, the method makes analysis cardinal consistency and semantics and also suggests, when necessary, the contour shape.

To facilitate the handling of the concepts of the MACBETH method, there are two software programs to implement and run the MACBETH method: the HiView software and the M-MACBETH software. The HiView software was chosen for the generation of arrays of judgment of the problem, to conduct several sensitivity analyses and robustness of the results of the model application, offering numerous graphical representations that facilitate the preparation of a report justifying the recommendations developed. Built from the MACBETH approach, the HiView tool is crucial for evaluation of models based on this method. Thus, the HiView software executes the functionalities of the M-MACBETH software, with the objective of verifying the consistency of the information and the potential of the methodology employed. Analyze the trend of local and global results of actions when it does vary replacement rates [24, 25]. The HiView provides decision-makers with professional confirmation of their judgments or even allows some values that are not in line with their expectations, validating the data model and consolidating the credibility of this being changed.

#### 3.4.4. Application of the Control Events into the Methodology to Support Decision-Making

**Figure 8:**
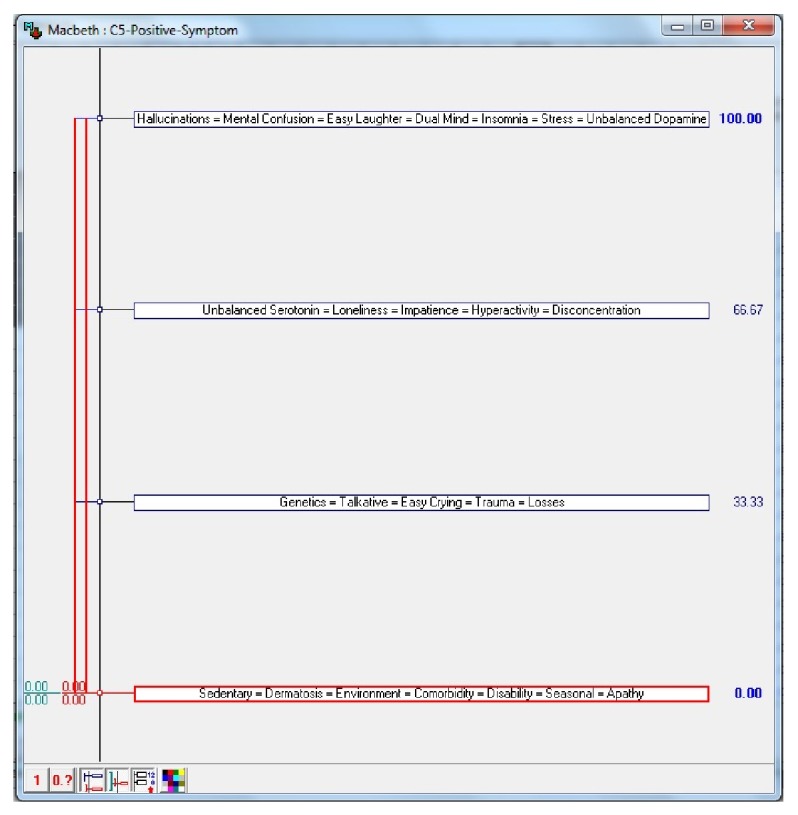
Control events with a new degree of confidence within allowed ranges. Source: formatted by the author from HiView software.

After undergoing methodology to support decision-making (MCDA—multicriteria decision aid), implemented in this work through HiView software, which also runs the MACBETH method, the “control events” retro mentioned, were adjusted so as to obtain the matrix of constant value judgment of Figures 6 and 7, which allows the visualization of degrees of attractiveness between events as well as the “current scale” of “confidence factors,” besides indicating if the results are coherent states “consistent judgments.” As for “confidence factors,” Figure 8 gives an idea of how the decision-maker can adjust these degrees of attractiveness between “control events,” pointing a ruler at the boundaries between the various degrees of attractiveness of these events. The new levels of attractiveness indicated on the ruler are reflected in the current range of the matrix as well as adjustments in the confidence of control events factors, as can be seen in Figure 8.

In the present work, the export of the control events and degrees of confidence will be made indirectly and through the collection of answers to questions correlated to each control event. That is, the control events analyzed using the MACBETH methodology, implemented in the HiView software, are associated with the variables treated by expert system, which can be of two quantities: common variables and objective variables. The common variables correspond to the symptoms and causes of the disorders studied. Already, the objective variables correspond to the final diagnosis to be found for the disorder.

Furthermore, as stated in the analysis of disorders seen in this study, each control event has a percentage of influence in relation to the disorder and in relation to the other control events. This percentage, derived from the scale discriminated in Table 1, will compose the degree of confidence in expert system, which will use the control event as an important part to the diagnosis. Thus, for each set of answers to the questions that appear in the user interface, the expert system links this set of responses to the degree of confidence of the control events in order to point the diagnosis. After constructing the array of value judgment, it is possible to do in the HiView software a sensitivity analysis as presented in Figure 8. This analysis allows changing the values of the degrees of confidence of the events of controls.

## 4. Specialist System Applied to the Diagnosis of Psychological Disorders

### 4.1. Specialist System: Contextualization

Expert systems are associated with the term artificial intelligence (AI). This expression was first used at a summer conference at Dartmouth College, USA, when researchers John McCarthy, Marvin Minsky, Nathaniel Rochester, and Claude Shannon met in order to conduct a study on this subject, which had been baptized with the expression that generated warm and controversial debates during the aforementioned conference and beyond it. Mentioned expression stayed, however, without a formal definition because, first of all, it was imperative to define, too, and formally, what intelligence would be. There are two main strands of research for the intelligent building systems: connectionist and symbolic. The first aims at the modeling of human intelligence by simulating the components of the brain, that is, their neurons and their interconnections. This proposal was first formalized in 1943, when the neuropsychologist McCulloch and, the logical, Pitts proposed a first logical mathematical model for a neuron. In turn, the second part followed the logic tradition and took McCarthy Newell and his principal investigators. Thus, knowledge-based systems, or expert systems, are built mainly with rules that reproduce the knowledge of experts in the fields of human knowledge and are used to solve particular problems in specific domains. The area of human health has been one of the most allotted portions by expert systems, due to being possessed by classic problems that need systems with these potentialities. It is important to note that there are characteristics that indicate whether a given problem should be solved by this information technology or not. To facilitate the process of analyzing a given problem, certain conditions must be met so that they can add value in identifying and choosing which expert systems technology as follows:

- Expert systems that require professional experts in knowledge related to the problem, considering that this knowledge will form the knowledge base that will be used in solving problems;
- activities to be performed that require the participation of groups of experts who, when alone, do not have sufficient knowledge to perform them;
- activities that require knowledge of the details that, if overlooked, cause degradation of performance;
- activities that show large differences in performance between experts from the group study the problem;
- number of experts available to solve the problem which is insufficient.

The criteria referenced in the previous paragraph provide important respects, for example, a significant increase in the productivity of a business decision maker, in performing specialized tasks when assisted by an intelligent system. It is noteworthy that the portability of these expert systems, being capable of developing and being used in microcomputers, is currently deciding factor. This characteristic causes these systems to become accessible and affordable. In general, systems with automated reasoning can be used banks incorporating existing data in the organization, or being incorporated into the set of tools available in the databases. Many work areas can be helped by structured expert systems solutions as these are efficient applications for information management. Providing tools to support decision-making in this case goes further than providing graphs and tables to the user, provide them a north in the identifying of their needs, simulating scenarios and allowing greater accuracy and reliability in the solutions of their problems. Expert systems are, therefore, computer programs that provide solutions to certain problems, in the same way that human experts offer under the same conditions. The most common architecture of expert systems involves production rules structuring simply a set of conditions in the IF… THEN… style, with the possibility of inclusion of logical connectives relating the attributes within the scope of knowledge and the use of probabilities.

### 4.2. The Expert SINTA

**Figure 9:**
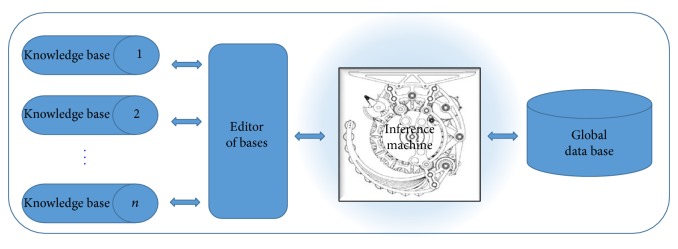
Simplified architecture of Expert SINTA. Source: adapted by the author from information of the LIA-UFC. Knowledge base comprises representative information of facts and rules that the expert uses. Editor of bases is the means by which the shell allows the implementation of desired knowledge bases. Inference machine is the part of the expert system responsible for logical deductions about the knowledge base. Global database consists of the evidence presented by the expert system user during a consultation.

The software Expert SINTA was created by a group of scholars at the Federal University of Ceará (UFC), and the State University of Ceará (UECE) called Group SINTA (Sistemas INTeligentes Aplicados or Applied Intelligent Systems) [26, 27]. It was developed using Borland Delphi technology. That is a computational tool that uses artificial intelligence techniques for automatic generation of expert systems. It uses a knowledge representation model based on production rules and probabilities, with the main objective of simplifying the construction work of expert systems through the use of a shared inference machine. The automatic construction of screens and menus, treatment of probabilistic rules production, and use of sensitive explanations to the context of the knowledge base modeled. An expert system based on this type of model is very useful in classification problems. The user responds to a sequence of menus, and the system provides answers that fit the framework identified by the user. Some of the main features of the Expert SINTA are use of backward chaining; use of confidence factors; tools for debugging; opportunity to include online help for each knowledge base. The expert systems are generated in the Expert SINTA using the architecture described in Figure 9.

**Figure 10:**
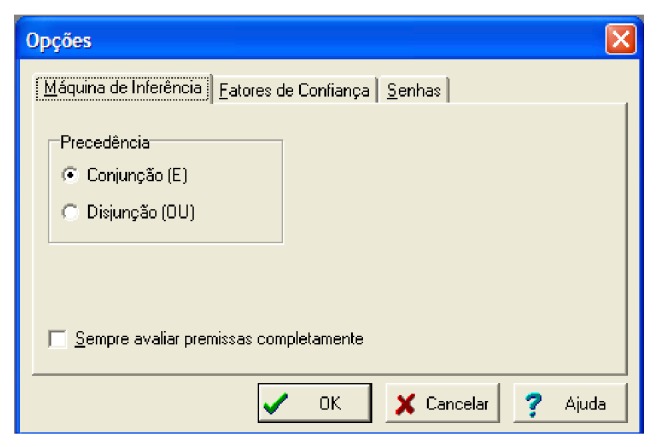
Precedence of logical operators. Source: formatted by the author from software Expert SINTA.

**Figure 11:**
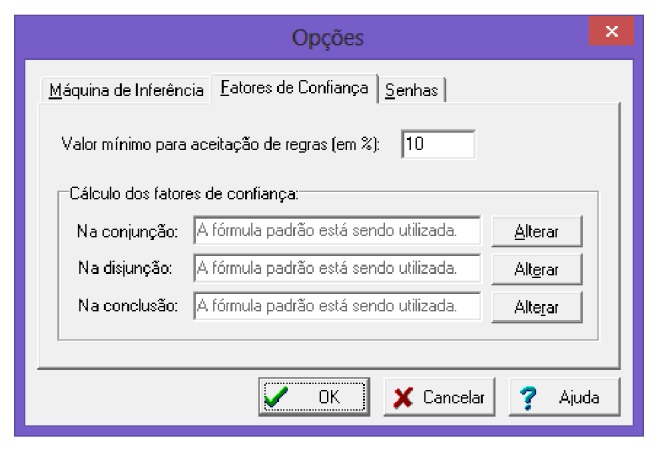
Setting minimum and indicative password. Source: formatted by the author from software Expert SINTA.

**Figure 12:**
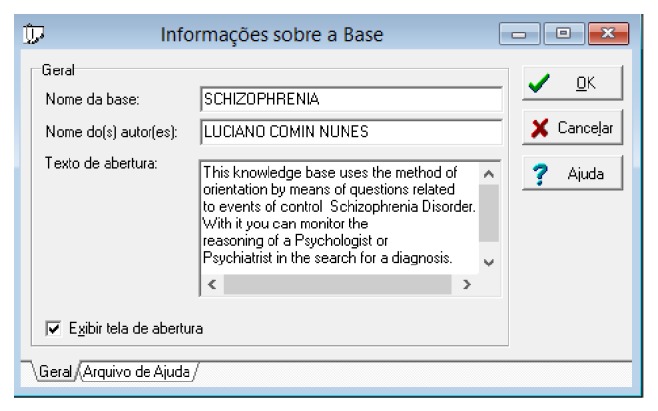
General information about the knowledge base of the expert system. Source: formatted by the author from software Expert SINTA.

**Figure 13:**
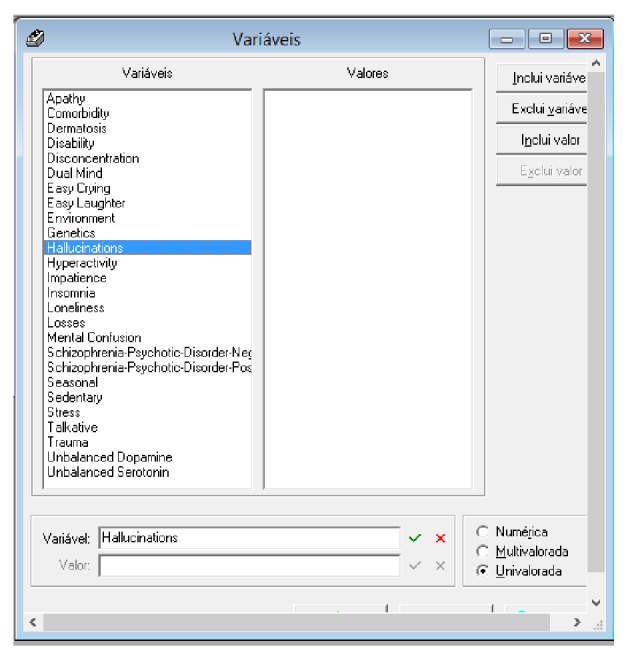
Definition of variables in Expert SINTA. Source: formatted by the author from software Expert SINTA.

**Figure 14:**
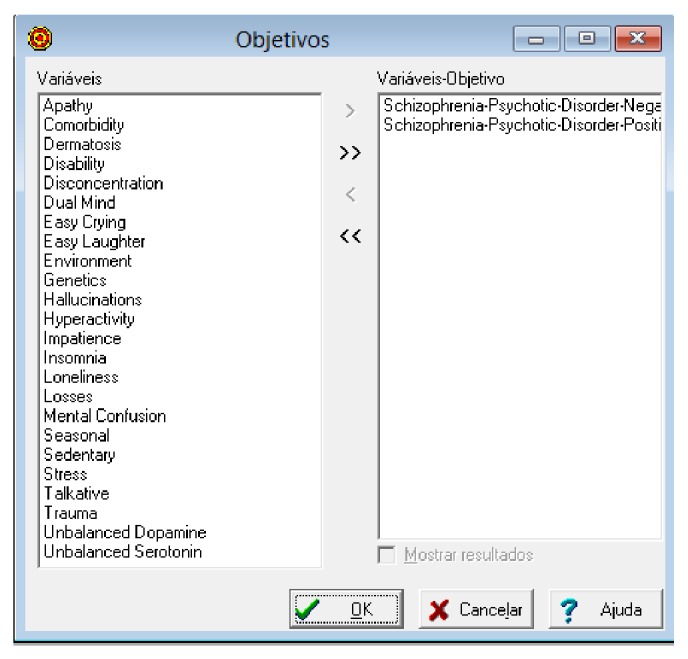
Definition of variables goal. Source: formatted by the author from software Expert SINTA.

**Figure 15:**
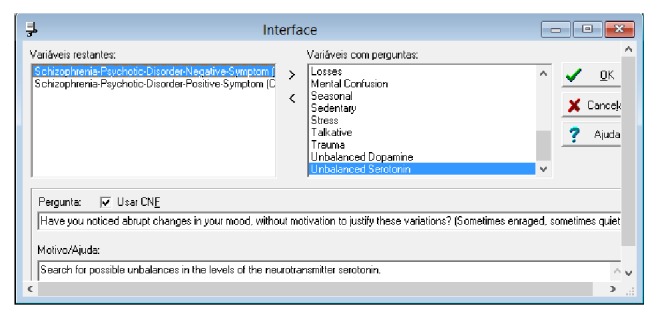
Creating user interfaces. Source: formatted by the author from software Expert SINTA.

**Figure 16:**
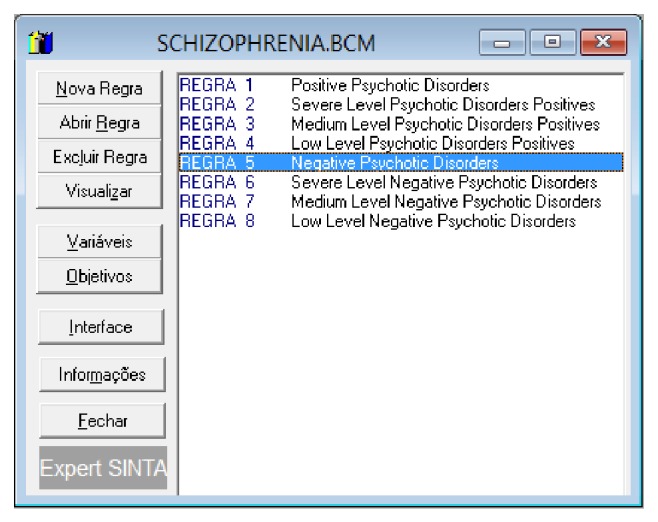
Creating logical rules. Source: formatted by the author from software Expert SINTA.

**Figure 17:**
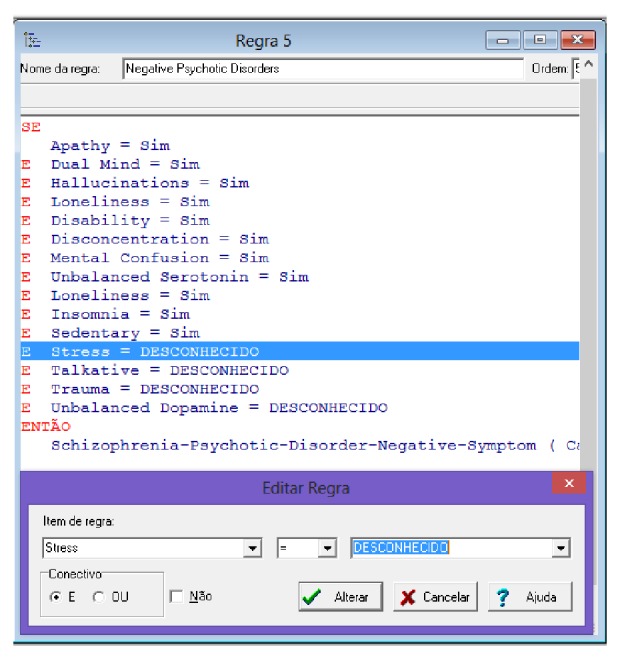
Definition of logical rules. Source: formatted by the author from software Expert SINTA.

**Figure 18:**
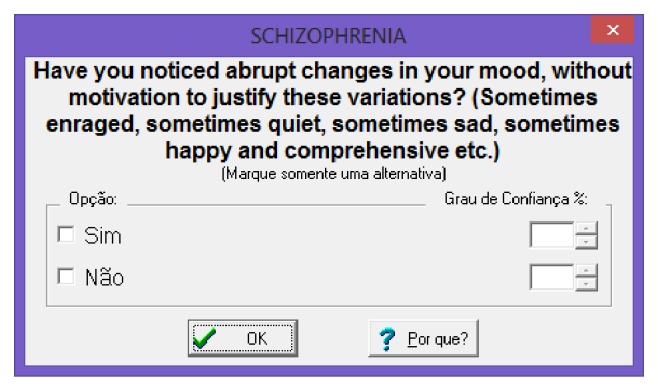
Input data through the user interface. Source: formatted by the author from software Expert SINTA.

**Figure 19:**
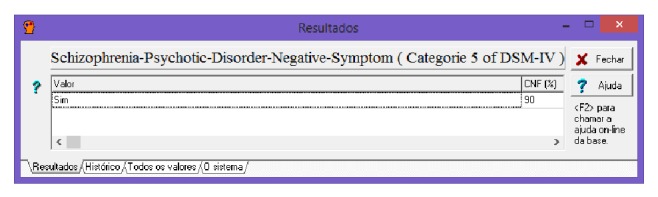
Possibility of diagnosis. Source: formatted by the author from software Expert SINTA.

The Expert SINTA aims to simplify the steps of creating a full expert system. For this, a basic inference engine is already offered, based on backward chaining. The backward chaining excels in problems where there are a large number of conclusions that can be achieved, but the number of ways in which they can be reached is not great, in problems where it cannot meet an acceptable number of facts before starting to search for answers. The backward chaining is also more intuitive for the developer, because it is based on recursion, an elegant and efficient way of programming, where the logic programming itself is directed. At no time, however, it ceases to recognize that the forward chaining has advantages in certain occasions. Moreover, the calculations of values of differences in degree of attractiveness and judgment needed to build the array of values generated by the MACBETH method, as shown in Figures 6 and 7. In Figures 10 to 19 the steps to building an expert system, with aim at assisting in the final diagnosis are presented. In Figure 10 there is an illustration of how the user in the Expert SINTA should proceed to set the precedence of the logical operators that will be used by the inference machine, that is, if (*A* and *B*) or *C*/*A* and (*B* or *C*).

The choice of the minimum value for the confidence factor is shown in Figure 11. Besides this, there is the possibility of placing a password for the database that will be created. This option is most commonly used as the basis of expert knowledge is confidential.

Figure 12 presents information about the knowledge base to be built. This window will appear after the user starts using the expert system.

On the other hand, Figure 13 shows how to define the variables in the Expert SINTA. These variables will feed the expert system knowledge base and, in the specific case, for each control event detailed variable will be created.

It is possible to see in Figure 14 the definition of objective variables, that is, those which point the final diagnosis in the expert system.

Moreover, Figure 15 shows how the interface is defined in the expert system for collecting events with respective degrees of confidence. For each variable, an issue that aims to collect symptoms of the disorder as well as a degree of confidence about this symptom felt by the patient is created.

At the time of creation of each rule, the Expert SINTA allows this rule to be inserted for desired preference. Moreover, Figure 16 shows the list of logical rules constructed from the rule editor of the Expert SINTA.

The editor of rules, which constitute the logical reasoning of the expert system, can be seen in Figure 17. Mentioned rules point the final diagnosis to the user of system. Note that the rules follow the following structure: IF… THEN… (SE… ENTÃO…).

During the execution of the expert system developed with Expert SINTA, the user can interact through graphical interfaces as shown in Figure 18. The interaction through these interfaces enables the collection of values, which will feed the variables and factors of trust used in the system specialist.

Figure 19 shows the diagnosis reached by the expert system after inferring the information supplied to him by the fed interfaces as well as preexisting knowledge bases in their information.

**Figure 20:**
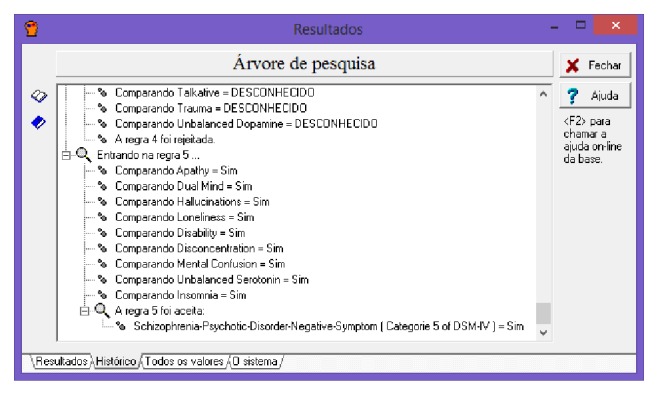
Trail to assist in analysis of outcomes after diagnosis. Source: formatted by the author from software Expert SINTA.

The Expert SINTA provides for each expert system the path of logical reasoning trod of the execution of the respective expert system.  Figure 20 provides an overview of this track, which helps in analyzing the results after obtaining a diagnosis.

## 5. Conclusion and Future Works

Many smart techniques were incorporated into the decision-making process. These methods are based on technologies that use concepts of artificial intelligence (AI), such as systems expert's genetic algorithms [26, 27], neural networks [28], intelligent agents, reasoning based on case studies [2, 29], and fuzzy logic [30].

Despite these technological advances, much more needs to be done to automate decision-making, especially when it involved a multicriteria analysis.

In the present study, we observed that the information generated in the multicriteria analysis methodology was used in the algorithm in order to transform them into variables with respective degrees of confidence, which were processed by the expert system inference engine through use of IF… THEN… rules, with the aim of using this information, pointing your diagnosis, incorporating it to their knowledge base. The automation of this process of transition between multicriteria methodology and expert system is a challenge that remains to be solved by the construction of a bridge that enables this integration. Unfortunately, such a task was not possible within this study, given the limited resources of time and availability of technical staff. Thus, the test performed in this study tried to show a hybrid model, using a connection manually. Therefore, we tried to present the feasibility of integration between the mentioned technologies: multicriteria methodology to support decision-making and expert system generated by Expert SINTA. With this proposal it is expected to have contributed to the quality of automated diagnosis.

Finally, it was realized that this field of work is still largely unexplored, fitting entities related to research, encouraging greatly having more concrete results, emphasizing compensatory be returns on that investment, because organizations have required based expert systems solutions.

It is suggested, therefore, in order to improve the model proposed in this paper, to take the following measures:

- Use of some other multicriteria methodologies, such as influence diagram and Bayesian networks;
- Improved interface with the user, the Expert SINTA including among its features the export and import of data files containing control events and degrees of confidence;
- Formatting and automation of generic questionnaires covering control events and degrees of confidence.

On the other hand, given that the psychiatry proves to be one of the specialties of medicine that formalized consciously and comprehensively the diagnostic process for the disorders of his study and domain and by virtue, the interest of the current* DSM-V* formulators, in categories and dimensional classification of mental disorders be compatible with neuroscientific and genetic foundations that itself want to consolidate, the authors plan in future work, include in the proposal hybrid information technology, beyond recommendations contained in the* DSM-V*, quantitative and dimensional model with image processing and scales of values, as a way to enhance the diagnostic process currently based on categorization of types of schizophrenia with events of control based in symptoms, causes and historical of this disease.

## Figures and Tables

**Table 1:** Range of influence of events in psychological disorders.

0	25	50	75	100
Indifference	Little influence	Moderate influence	Much influence	Decisive influence

Source: idealized by the author from reports of experts in psychiatry and psychology.

**Table 2:** Decision matrix.

Criteria→	*g*_1_	*g*_2_	⋯	*g*_*j*_	⋯	*g*_*n*_
Limit→	*q*_1_, *p*_1_	*q*_2_, *p*_2_	⋯	*q*_*j*_, *p*_*j*_	⋯	*q*_*n*_, *p*_*n*_

Alternatives↓						
*A*_1_	*a*_11_	*a*_12_	⋯	*a*_1*j*_	⋯	*a*_1*n*_
*A*_2_	*a*_21_	*a*_22_	⋯	*a*_2*j*_	⋯	*a*_2*n*_
⋮	⋮	⋮	⋮	⋮	⋮	⋮
*A*_*i*_	*a*_*i*1_	*a*_*i*2_	⋯	*a*_*ij*_	⋯	a_in_
⋮	⋮	⋮	⋮	⋮	⋮	⋮
A_m_	a_m1_	a_m2_	⋯	a_mj_	⋯	a_mn_

Source: [6].
